# THE ASSOCIATION BETWEEN PHYSICAL ACTIVITY AND NECK CIRCUMFERENCE WITH CARDIOVASCULAR DISEASE RISK IN OLDER WHEELCHAIR USERS

**DOI:** 10.2340/jrm.v56.35279

**Published:** 2024-06-19

**Authors:** Jeonghyeon KIM, Inhwan LEE, Hyunsik KANG

**Affiliations:** 1 College of Sport Science, Sungkyunkwan University, Seoul; 2 Department of Antiaging and Health Care, College of Future Convergence, Changwon National University, Changwon, Republic of Korea

**Keywords:** disability, older adults, cardiovascular disease, physical activity, neck circumference

## Abstract

**Objective:**

To examine the association between physical activity, neck circumference, and cardiovascular disease risk in older wheelchair users.

**Design:**

A cross-sectional study.

**Subjects/Patients:**

Sixty-one Korean wheelchair users aged 50 years and older.

**Methods:**

Physical activity was assessed using a self-administered questionnaire. Neck circumference was measured with a tape ruler. Cardiovascular disease risk was evaluated by calculating the Framingham risk score (FRS) for estimating 10-year cardiovascular disease risk, which was classified as low–moderate (19% or less) or high risk (20% or more).

**Results:**

The FRS for 10-year cardiovascular disease risk was inversely related to physical activity (beta [SE] = –0.213 (0.103), *p* = 0.043) and positively related to neck circumference (beta [SE] = 1.331 ± 0.419, *p* = 0.003). Binary logistic regression showed that those with low physical activity (odds ratio [95% confidence interval] = 4.256 (1.188~15.243), *p* = 0.026) or a large neck circumference (odds ratio [95% confidence interval] = 3.645 (1.172~11.338), *p* = 0.025) had a higher risk for high cardiovascular disease risk compared with those with high physical activity or normal neck circumference.

**Conclusion:**

The current study findings suggest that an intervention targeting physical inactivity and upper-body obesity should be implemented to reduce cardiovascular disease risk in older wheelchair users.

LAY ABSTRACT

This study looked at how physical inactivity and upper-body obesity affect the risk of cardiovascular disease in a small group of wheelchair users recruited from local sports centres. In this study, we found that a lack of physical activity as well as a large neck circumference were associated with an increased risk of cardiovascular disease. We discovered that those who do not exercise regularly or have a large neck circumference are more likely to develop cardiovascular disease than those who exercise regularly or have a normal neck circumference. The findings of the study emphasize the importance of engaging in physical activity and/or reducing upper-body obesity to reduce the risk of cardiovascular disease incidence in people with disabilities.

As of 2016, 2.511 million people in South Korea were registered as disabled, and more than half of those were aged 65 years and older. Physical disability accounted for 50.5% of the total, followed by 11.6% for speech/hearing, and 10.1% for visual disability. Approximately 6.2% of disabled people require the use of a wheelchair due to their limited or insufficient walking ability (1). As a result, wheelchair users are less active and more sedentary than able-bodied people (2). This is especially true for older wheelchair users, who may face additional physical challenges as well as potential barriers in physical and socio-cultural settings (3).

Cardiovascular disease (CVD) is the leading cause of death in the world, accounting for nearly one-third of all-cause deaths (https://www.who.int/health-topics/cardiovascular-diseases#tab=tab_1). South Korea is experiencing a similar situation (4). Wheelchair users are especially vulnerable to hypokinetic diseases such as CVD (5), as well as premature death from CVD (6). Individuals with physical disability were at higher risk of developing CVD in a 7.5-year retrospective cohort study involving 6,419 disabled adults (7). A nationwide longitudinal study of 514,679 Korean adults found that disabled individuals had a higher risk of CVD incidence and mortality over a mean follow-up of 10.8 ± 3.9 years (8). Modifiable lifestyle risk factors such as smoking, high blood pressure, high cholesterol, diabetes, overweight and obesity, physical inactivity, poor nutrition, and heavy alcohol consumption account for a substantial portion of the incidence and prevalence of CVD (9).

Neck circumference (NC) is indicative of upper body obesity, and it is an accurate predictor of CVD risk in both older adults (10) and CVD patients (11). For example, neck circumference is significantly and positively related to incident atrial fibrillation (12) and cerebrovascular disease (13). NC is also linked to CVD in hypertensive patients (11), as well as CVD events and mortality in a high-risk population (14). However, little is known about the relationship between NC and CVD risk in wheelchair users. Physical inactivity is another independent risk factor for CVD and the fourth leading risk factor of all-cause mortality (15). As a countermeasure, the World Health Organization has issued recommendations encouraging disabled people including wheelchair users to engage in regular physical activity (16). Wheelchair users due to physical disability can benefit from alleviating many health conditions by increasing even minor levels of physical activity (17).

The Framingham Heart Study data was used to develop Framingham risk score (FRS) algorithms for estimating 10-year CVD. The gender-specific FRS considers 6 coronary risk factors: age, total cholesterol (TC), high-density lipoprotein cholesterol (HDLC), systolic blood pressure (SBP), diabetes, and smoking (18). The FRS has been well validated as a CVD risk prediction tool in various populations (19, 20). The FRS algorithms, however, do not consider the impact of NC and physical inactivity as exposures, especially in people with disabilities. This study aimed to investigate the association between physical activity, neck circumference, and CVD risk in older wheelchair users.

## METHODS

### Data source and study participants

**Fig. 1:**
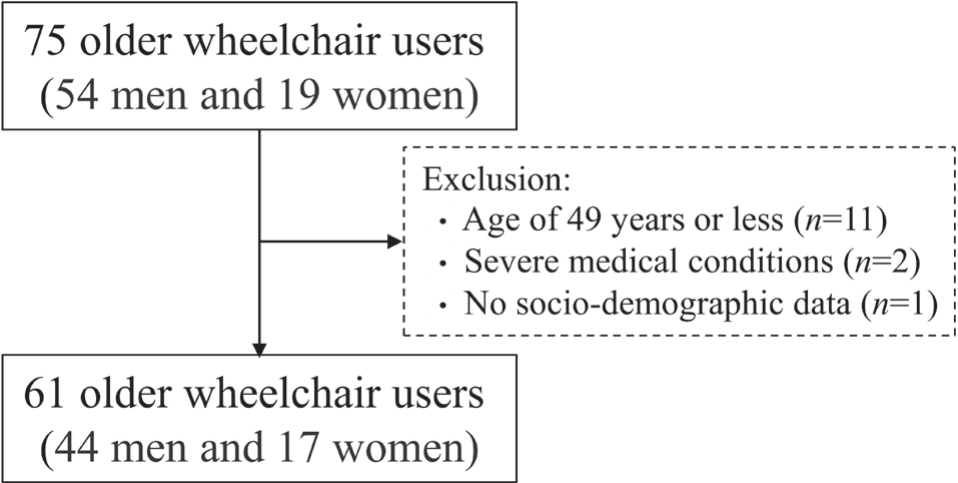
Selection procedure of study participants.

In a cross-sectional study design, as illustrated in Fig. 1, we recruited 75 participants (54 men and 19 women) from local community sports centres for people with disabilities in Gyeonggi-do and Chungcheong-do, Republic of Korea. Inclusion criteria included the age of 50 years and over and requiring a wheelchair due to a physical disability. Any medical complication preventing study participation was a criterion for exclusion. Ages 49 and younger (*n* = 11), severe medical complications (*n* = 2), and lack of demographics and data availability (*n* = 1) were all excluded. The remaining 61 participants (44 men and 17 women) were included in the final data analysis. Written informed consent was obtained from all participants before they participated in the study. The study protocols were reviewed and approved by the institutional review board (approval number 1040191-202210-HR-008-01).

### Body composition and cardiovascular risk factors

Height, weight, and blood pressure were assessed according to standardized protocols. Height was measured with a tape ruler while lying down on a floor, and weight was measured using a portable weight scale (AD-5105NP, Bucheon, Korea). Body mass index (BMI) was calculated as weight divided by height (kg/m^2^). Waist circumference (WC) and NC were measured with a tape ruler placed between the top of the hip bone and the bottom of the ribs (21) and at the level just below the laryngeal prominence perpendicular to the long axis of the neck with the head positioned in the Frankfurt horizontal plane (10), respectively. NC was then classified as normal or large based on the group’s median value of 38 cm. Total body composition including lean and fat mass was measured using a body composition analyser (S10, Inbody, Seoul, Korea). Resting blood pressure was measured using a portable sphygmomanometer (OMRON-M5, OMRON Healthcare, Dalian, China) in a seated position, with the arm at heart level and resting on the armrest of a chair. Fasting blood concentrations of glucose, TC, triglycerides (TG), and HDLC were measured using the LABGEO PT10 blood chemistry analyser (Samsung Electronics, Seoul, Korea).

### Physical activity

The Korean Physical Activity Scale for Individuals with Physical Disabilities (K-PASIPD), which consists of 13 questions (22), was used to collect leisure, household, and occupational activities in person (23). The validity and reliability of the K-PASID were previously tested in Korean older adults (23). Total physical activity was expressed in METs-h/day and classified as low or high based on the group’s median value.

### Cardiovascular disease risk

The CVD risk was assessed by estimating the 10-year FRS for CVD, which was calculated using the 1988 Framingham risk factor criteria and gender-specific algorithms based on age, TC, HDLC, SBP, and smoking status (18). The 10-year FRS percentage for CVD was low risk (< 10%), moderate risk (10–19%), and high risk (20+%). We redefined the 10-year FRS percentage for CVD as low–moderate risk (19% or less) and high risk (20% or more), combining low risk and moderate risk into low–moderate risk.

### Demographics

Age (years), gender (male vs female), education (elementary/less, middle/high school, or college/better), monthly income (in Korean won), smoking (past/current smoker vs non-smoker), heavy alcohol consumption (7 drinks per week for males or 5 drinks per week for females), and injury statistics (i.e., duration, cause, and type) were all measured using self-administered questionnaires.

### Statistical analyses

Quantile–quantile plots were used to verify the normality of the data distribution before statistical analyses. Analysis of variance (ANOVA) and a chi-square test were used to test mean group differences of continuous (in mean and standard deviation) and discontinuous (in number and percentage) variables, respectively. Multicollinearity refers to how much information is shared between variables, making it difficult to determine how much each variable influenced the regression. We detected multicollinearity by calculating the variance inflation factor (VIF) and excluding variables with a VIF greater than 4. Multivariate linear regression was used to calculate the beta coefficients of the dependent variables with a VIF smaller than 4 for CVD risk. Finally, binary logistic regression was then used to calculate the odds ratio (OR) and 95% confidence interval (CI) of high CVD risk by physical activity (PA) and NC levels. Statistical significance was tested at *p* = 0.05 using SPSS-PC software (version 27, IBM Corp, Armonk, NY, USA).

## RESULTS

Sixty-one of the 71 initial participants (81% acceptance rate) finished all the measurements required for the study. As shown in Table I, men and women had similar average ages of 59.3 ± 5.6 and 61.2 ± 5.1 years, respectively. Men had shorter injury duration (*p* = 0.033) and lower body fat (*p* < 0.001), but had larger NC (*p* < 0.001), higher skeletal muscle mass (*p* < 0.001), and higher FRS for estimating 10-year CVD risk (*p* = 0.003) compared with women. There were no gender differences in education, marital status, cause of disability, types of disability, health behaviours (i.e., drinking, number of medications, and physical activity), BMI, fat mass, WC, and individual CVD risk factors. Additionally, there was a decremental linear trend in PA (*p* = 0.032) but an incremental linear trend in BMI (*p* = 0.028), WC (*p* < 0.001), and NC (*p* = 0.008) according to CVD risk levels: the higher the CVD risk, the less physically active, and the heavier and more centrally obese (Table SI).

**Table I:** Physical characteristics of study participants

Variables	Total (*n* = 61)	Men (*n* = 44)	Women (*n* = 17)	*p*-value
Socio-demographics				
Age (years)	59.8 ± 5.5	59.3 ± 5.6	61.2 ± 5.1	0.212
Education, *n* (%)				0.427
Elementary or less	6 (9.8)	3 (6.8)	3 (17.6)	
Middle/high school	50 (82.0)	37 (84.1)	13 (76.5)	
College or better	5 (8.2)	4 (9.1)	1 (5.9)	
Income (10,000 won/month)	227 ± 156	220 ± 144	244 ± 188	0.607
Marital status, *n* (%)				0.341
Married	47 (77.0)	33 (75.0)	14 (82.4)	
Divorced/widowed	9 (14.8)	6 (13.6)	3 (17.6)	
Unmarried	5 (8.2)	5 (11.4)	0 (0)	
Injury statistics				
Duration of injury (years)	33.5 ± 18.3	30.5 ± 17.3	41.5 ± 18.9	0.033
Cause of disability, *n* (%)				0.052
Congenital	3 (4.9)	2 (4.5)	1 (5.9)	
Birth injuries	2 (3.3)	0 (0)	2 (11.8)	
Diseases	18 (29.5)	11 (25.0)	7 (41.2)	
Accidents	38 (62.3)	31 (70.5)	7 (41.2)	
Types of disability, *n* (%)				0.071
Poliomyelitis	16 (26.2)	8 (18.2)	8 (47.1)	
Spinal cord injury	37 (60.7)	30 (68.2)	7 (41.2)	
Myelitis	3 (4.9)	2 (11.8)	1 (2.3)	
Arthritis	1 (1.6)	1 (2.3)	0 (0)	
Brain lesions	4 (6.6)	4 (9.1)	0 (0)	
Health behaviours				
Alcohol consumption, *n* (%)	9 (14.8)	7 (15.9)	2 (11.8)	0.682
Number of medications	1.2 ± 1.1	1.3 ± 1.1	1.2 ± 1.0	0.817
Physical activity (METs-h/d)	23.5 ± 9.8	22.9 ± 9.7	24.9 ± 10.1	0.482
Body composition				
Body mass index (kg/m^2^)	25.8 ± 4.3	25.8 ± 4.2	25.6 ± 4.7	0.871
Skeletal muscle mass (kg)	19.5 ± 4.9	21.2 ± 4.5	15.3 ± 3.0	< 0.001
Fat mass (kg)	29.1 ± 8.2	28.8 ± 8.0	29.8 ± 8.9	0.649
Body fat (%)	43.5 ± 8.1	41.5 ± 8.1	48.8 ± 5.0	< 0.001
Waist circumference (cm)	91.9 ± 12.2	93.6 ± 11.7	87.7 ± 12.8	0.089
Neck circumference (cm)	38.5 ± 3.8	39.6 ± 3.4	35.6 ± 3.4	< 0.001
CVD risk factors				
TG (mg/dL)	172.3 ± 89.7	159.5 ± 86.9	205.5 ± 90.9	0.072
TC (mg/dL)	166.1 ± 41.7	160.3 ± 37.6	18.8 ± 48.8	0.085
LDLC (mg/dL)	80.6 ± 39.6	79.0 ± 38.8	84.8 ± 42.5	0.612
HDLC (mg/dL)	50.7 ± 11.3	49.1 ± 9.3	54.9 ± 14.8	0.070
SBP (mmHg)	141.8 ± 16.2	141.7 ± 17.7	141.9 ± 12.1	0.965
SBP treated, *n* (%)	24 (39.3)	17 (38.6)	7 (41.2)	0.856
Diabetes, *n* (%)	19 (31.1)	14 (31.8)	5 (29.4)	0.855
Smoking, *n* (%)	12 (19.7)	11 (25.0)	1 (5.9)	0.092
FRS for 10-year CVD risk (%)	17.6 ± 7.9	19.4 ± 7.5	12.8 ± 7.0	0.003

FRS: Framingham risk score; CVD: cardiovascular disease; TG: triglyceride; TC: total cholesterol; LDL-C: low-density lipoprotein cholesterol; HDL-C: high-density lipoprotein cholesterol; SBP: systolic blood pressure.

**Fig. 2:**
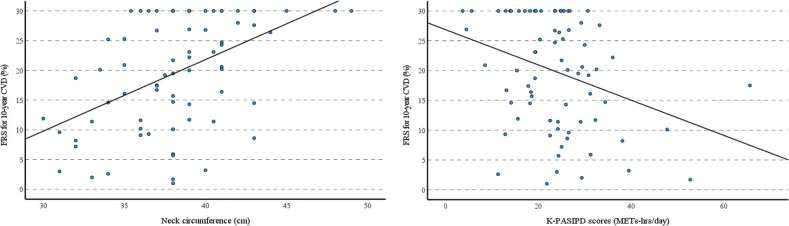
Illustration of the relationship between neck circumference and physical activity. Physical activity was measured using the Korean Physical Activity Scale for Individuals with Physical Disabilities (K-PASIPD) in METs-hours per day. FRS: Framingham risk score; CVD: cardiovascular disease.

We performed a multivariate linear regression to determine predictors of the FRS for 10-year CVD. As shown in Table II, PA (unstandardized β = –0.270, *p* = 0.013) and NC (unstandardized β = 1.253, *p* = 0.004) were significant determinants of the estimated CVD risk in this study population. As shown in Fig. 2, the FRS for 10-year CVD was positively related to NC and inversely to PA.

**Table II:** Multiple linear regression for the Framingham risk score to estimate 10-year cardiovascular disease

Variables	Unstandardized β (SE)	Standardized β	*p*-value	VIF
Physical activity	–0.270 (0.103)	–0.327	0.013	1.167
Neck circumference	1.253 (0.403)	0.551	0.004	2.339
Skeletal muscle mass	–0.036 (0.339)	–0.020	0.917	2.730
Marital status	–1.653(1.740)	–0.130	0.347	1.325
Educational background	1.872 (2.887)	0.080	0.521	1.143
Monthly income	–0.004 (0.005)	–0.086	0.503	1.209
Duration of disability	0.079 (0.068)	0.156	0.257	1.360
Type of disability	–0.401 (1.444)	–0.043	0.783	1.760
Alcohol consumption	2.329 (1.526)	0.221	0.136	1.558

VIF: variance inflation factor.

Finally, we conducted a binary logistic regression analysis to calculate ORs and 95% CIs for the FRS-based high CVD risk according to PA and NC levels. As shown in Table III, people with low PA were more likely to have high CVD risk (OR = 3.429, 95% CI = 1.152~10.202, *p* = 0.027) compared with people with high PA (OR = 1). The increased high CVD risk remained statistically significant (OR = 4.256, 95% CI = 1.188~15.243, *p* = 0.026) even after adjusting for NC. Furthermore, people with high NC were more likely to have high CVD risk (OR = 3.810, 95% CI = 1.275~11.385, *p* = 0.017) compared with people with low NC (OR = 1). The increased high CVD risk remained significant (OR = 3.645, 95% CI = 1.172~11.338, *p* = 0.025) even after adjusting for PA.

**Table III:** Odds ratios (95% confidence intervals) of physical activity and neck circumference for high cardiovascular disease (CVD) risk

Variables		High CVD risk			
		Model 1	*p*-value	Model 2	*p*-value
Physical activity	High	1		1	
	Low	3.429 (1.152 ~ 10.202)	0.027	4.256 (1.188 ~ 15.243)	0.026
Neck circumference	Normal	1		1	
	Large	3.810 (1.275 ~ 11.385)	0.017	3.645 (1.172 ~ 11.338)	0.025

Model 1: unadjusted.

Model 2: The OR for low physical activity was adjusted for education, monthly income, marital status, and neck circumference, and the OR for high neck circumference was adjusted for education, monthly income, marital status, and physical activity.

High CVD risk was defined as 20% or more Framingham risk score for estimating 10-year CVD risk.

Physical activity and neck circumference were classified as low or high and normal or large, respectively, based on their median values.

## DISCUSSION

In this study, we investigated the association between PA, NC, and CVD risk in 61 Korean wheelchair users aged 50 years and older. The current findings of the study suggest that older wheelchair users who are physically inactive and/or have a large NC are at a higher risk of CVD. To the best of our knowledge, this is the first study to show that, along with physical inactivity, a large NC is significantly and positively related to CVD risk in older wheelchair users.

The current findings apply to all older wheelchair users, not just Koreans. For example, the inverse relationship between PA and CVD risk observed in this study has been observed in people without disabilities (24), and the association is reviewed and summarized in a meta-analysis study of randomized controlled trials and longitudinal studies (25). Similarly, physical inactivity is a major contributor to poor health outcomes in people with disabilities (26). Older wheelchair users are especially vulnerable to hypokinetic diseases such as CVD due to limited or inadequate mobility (27) and/or additional physical challenges and physical and socio-cultural barriers to PA (3). Taken together, the findings of the current and previous studies highlight the urgency of encouraging PA for wheelchair users to reduce their CVD burden and improve their overall well-being (17).

The positive association between NC and CVD risk observed in the current study has been reported in previous studies involving people without disabilities. In the Framingham studies, NC was positively and independently associated with CVD risk factors (28) and incident atrial fibrillation (12). In a 7.6-year follow-up study, Hu et al. (10) reported that a large NC at baseline was a significant predictor of CVD events in community-dwelling Chinese older adults. In a clinical study of 232 atherosclerotic disease patients who were admitted to outpatient clinics, Asil et al. (29) showed that a larger NC was significantly related to the estimated systematic coronary risk. In the Brazilian Longitudinal Study of Adult Health, Almeida-Pititto et al. (30) showed that a large NC was significantly associated with unfavourable atherogenic metabolic profiles in middle-aged individuals who were at low cardiovascular risk levels. Additionally, NC is predictive of upper body obesity in a multicentre study of 8 Latin American countries (31), metabolic syndrome in a population-based study of Korean adults (32), and chronic kidney disease in 177 consecutive patients who were at high CVD risk (33), as well as all-cause mortality and heart failure hospitalization in African Americans (34). Taken together, the findings of the current and previous studies suggest that NC could be used as a novel biomarker in determining CVD risk in older wheelchair users.

There are several explanations for the current findings concerning the relationship between PA, NC, and CVD risk in older wheelchair users. First, physical inactivity has several CVD risk factors, such as elevated blood pressure, unfavourable lipoprotein profiles, decreased insulin sensitivity, inflammation, decreased endothelial function, arterial wall stiffness, impaired angiogenesis, impaired autonomic function, and others (35), all of which contribute to increased CVD risk. Second, a large NC is associated with a clustering of metabolic risk factors, such as impaired fasting and postprandial homeostasis, atherogenic lipoprotein profiles, elevated blood pressure, endothelial dysfunction, inflammation, and others, all of which contribute to increased CVD risk (28). Third, a lack of PA combined with a large NC is more likely to amplify each risk factor for CVD, particularly in older wheelchair users.

To the best of our knowledge, this is the first study to show that physical inactivity, central obesity, and living alone are significant predictors of CVD in older disabled wheelchair users in Korea. This study has some limitations. First, the cross-sectional nature of the study precludes us from providing a cause-and-effect explanation for the association between PA, NC, and CVD risk. Second, because the sample size is small in comparison with the number of covariates, we cannot rule out the possibility of overfitting in multivariate regression analysis. Third, the small sample size of the current study limits the generalization of the findings. As a result, the current findings must be confirmed in a larger sample study before they can be applied to older wheelchair users.

In conclusion, given the aetiologic link between physical inactivity and a large NC and increased CVD risk in different populations (36), the current study findings indicate that a therapeutic strategy focusing on physical activity and a healthy diet should be implemented for older disabled wheelchair users.

## Supplementary Material

THE ASSOCIATION BETWEEN PHYSICAL ACTIVITY AND NECK CIRCUMFERENCE WITH CARDIOVASCULAR DISEASE RISK IN OLDER WHEELCHAIR USERS
