# Supplementary material for: THE ASSOCIATION BETWEEN PHYSICAL ACTIVITY AND NECK CIRCUMFERENCE WITH CARDIOVASCULAR DISEASE RISK IN OLDER WHEELCHAIR USERS
Source: J Rehabil Med. 2024 Jun 19;56:35279. doi: 10.2340/jrm.v56.35279 (PMC11218756; doi:10.2340/jrm.v56.35279)
Supplement: THE ASSOCIATION BETWEEN PHYSICAL ACTIVITY AND NECK CIRCUMFERENCE WITH CARDIOVASCULAR DISEASE RISK IN OLDER WHEELCHAIR USERS [file JRM-56-35279-s1.pdf]

Supplementary material has been published as submitted. It has not been copyedited, or typeset by Journal of Rehabilitation Medicine

Table SI. Health behaviors and body composition according to Framingham risk score for 10-year CVD risk

Variables	Low risk (*n*=13)	Moderate risk (*n*=26)	High risk (*n*=22)	*p* for linear trend
Health behaviors				
Heavy alcohol intake, n (%)	2 (15.4)	4 (15.4)	3 (13.6)	0.874
Number of medications	1.1±1.1	1.0±1.0	1.5±1.2	0.282
Physical activity (METs-hr/d)	27.3±8.4	24.4±11.5	20.2±7.3	0.032
Body composition Parameters				
BMI (kg/m^2)	24.2±3.2	25.2±4.5	27.4±4.4	0.028
Skeletal muscle mass (kg)	17.9±5.7	19.5±4.7	20.6±4.7	0.130
Fat mass (kg)	26.6±6.9	28.5±8.6	31.2±8.2	0.098
Body fat (%)	43.7±6.9	43.0±9.0	44.0±7.7	0.846
Waist circumference (cm)	85.8±9.6	90.9±12.1	96.7±12.2	0.008
Neck circumference (cm)	35.9±4.0	38.0±2.6	40.6±4.0	<0.001

BMI: body mass index; CVD: cardiovascular disease.
